# Research Progress in Multidimensional Prediction of Machining-Induced Surface Residual Stress

**DOI:** 10.3390/ma19030510

**Published:** 2026-01-27

**Authors:** Zichuan Zou, Xinxin Zhang, Wei Gong

**Affiliations:** School of Mechanical & Electrial Engineering, Guizhou Normal University, Guiyang 550025, China

**Keywords:** residual stress, empirical model, analytical model, finite element model, hybrid model

## Abstract

Intense thermo-mechanical coupling effects during cutting generate residual stress within the surface layer of a workpiece. This residual stress is a critical factor influencing the fatigue life, corrosion resistance, and dimensional stability of mechanical components, making its accurate prediction and control essential for improving product performance. To address the often generalized treatment of residual stress prediction modeling in existing literature, this paper presents a systematic review of recent advances in surface residual stress prediction for cutting operations. It details the formation mechanisms and significance of residual stress, focusing on four primary modeling approaches: empirical models based on experimental data, analytical models founded on metal cutting and elastoplastic theory, finite element models that simulate actual machining conditions, and hybrid models. A comprehensive analysis and comparison of these four model types is provided, summarizing their respective advantages and limitations. Furthermore, this paper identifies potential future research directions and development trends in residual stress prediction modeling, serving as a valuable reference for work in this field.

## 1. Introduction

Machining, as one of the most fundamental and widely used material forming processes in manufacturing, directly governs the in-service performance and reliability of components via the resultant surface integrity. Surface residual stress induced by machining is an internal, self-equilibrating stress field within the material, arising from non-uniform plastic deformation, thermomechanical coupling, and phase transformation effects during the machining process [1]. This stress state exerts a significant influence on key component properties, including fatigue life [2], resistance to stress corrosion cracking [3], and dimensional stability [4,5]. Driven by the increasingly stringent surface integrity requirements for high-end equipment such as aero-engine blades and nuclear reactor components [6,7], the field of residual stress prediction has progressed from traditional experimental characterization towards an integrated intelligent framework that combines numerical simulation, analytical modeling, and data-driven techniques.

Residual stress has both beneficial and detrimental effects and is primarily classified into two types: residual compressive stress and residual tensile stress. Residual compressive stress can effectively inhibit the initiation and propagation of fatigue cracks, thereby significantly improving a component’s fatigue life, corrosion resistance, and resistance to stress corrosion cracking [8]. Conversely, residual tensile stress tends to accelerate fatigue failure, diminish the load-bearing capacity of parts, and can even induce premature component failure under extreme conditions [9]. Additionally, a non-uniform residual stress distribution constitutes a primary cause of warping or distortion in precision-machined components such as thin-walled parts [10]. Therefore, the accurate prediction and active control of machining-induced residual stress has emerged as a critical technical challenge in advancing the surface quality and in-service reliability of high-end equipment. From a modeling perspective, tensile and compressive residual stresses are not only categories but also reflect different dominant mechanisms. Therefore, a practical model should be assessed by whether it can reproduce (i) the stress sign at/near the surface, (ii) the depth-wise transition, and (iii) the sensitivity to process variables. Empirical/Machine learning models can capture the sign and trend within the training domain but may suffer from limited extrapolation. Analytical models offer interpretability by separating thermo-mechanical contributions but rely on simplifying assumptions. Finite Element Model (FEM) provides the most complete field prediction at the cost of higher computational requirements and greater sensitivity to constitutive, contact, and mesh settings. Hybrid approaches aim to retain physics-based fidelity while reducing computational cost and enabling parameter identification [11].

Residual stress can be determined through two primary approaches: experimental measurement and model prediction. The former relies on techniques like X-ray diffraction [12] or the hole-drilling method [13] for direct measurement. Although reliable, these experimental methods are often costly, time-intensive, and typically limited to discrete sampling, posing challenges for comprehensive process optimization and real-time monitoring [14]. The latter approach constructs statistical or simulation models to elucidate the relationships between residual stress and influencing factors, such as cutting operations, tool geometry, cutting parameters, and material properties, thereby mitigating the difficulties associated with assessing residual stress in challenging environments. Recent research on machining-induced surface residual stress has predominantly centered on its effects on surface properties, underlying formation mechanisms, measurement techniques, and general influencing trends.

In contrast, dedicated and systematic reviews focusing specifically on predictive modeling methodologies remain comparatively scarce [11,15,16]. Despite prediction models being recognized as a crucial tool in residual stress research and attracting considerable interest, a thorough analysis and synthesis of these modeling techniques for machining applications is still lacking. Consequently, a systematic review of residual stress modeling methods is of considerable research significance and value.

**Figure 1:**
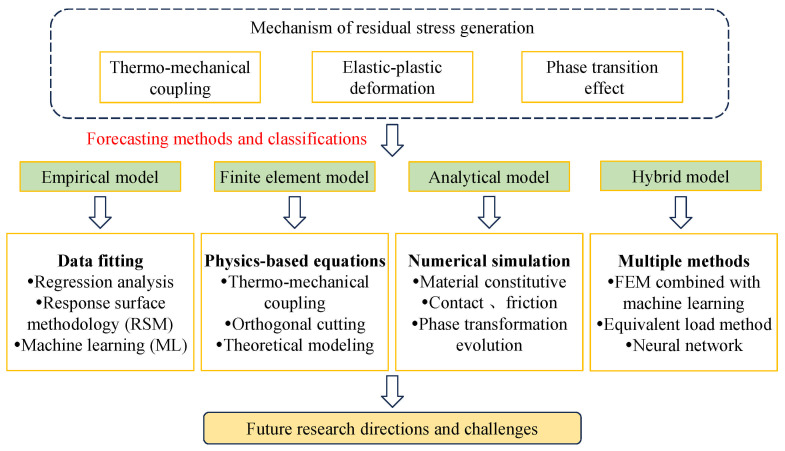
Flowchart of research progress in the prediction of residual stress.

From an application-oriented perspective, residual stress prediction models are not only evaluated by their theoretical formulation, but also by their ability to reproduce experimentally measured stress profiles and to support industrial decision-making. In practice, empirical and data-driven models are widely used for rapid trend analysis and parameter optimization, where experimental techniques such as X-ray diffraction or hole-drilling methods provide reference data for model calibration. Modeling methods for predicting residual stress are generally classified into four categories: empirical, analytical, finite element (FE), and hybrid modeling. To provide a systematic overview of the advancements in this field, this paper presents an analysis and discussion of these four mainstream approaches for predicting surface residual stress in machining. It details the fundamental principles, merits, and limitations of each method, as well as recent developments associated with them, and proposes potential future research directions. The overall structure and flow of this review are outlined in the research roadmap shown in Figure 1.

## 2. Residual Stress Prediction Methods

### 2.1. Empirical Model

Empirical models for predicting residual stress rely primarily on extensive experimental data and empirical correlations, as opposed to rigorous, physics-based numerical models. Substantial research indicates that cutting parameters [17,18,19,20], workpiece material [21], tool geometry, and tool wear [22] profoundly influence the magnitude and distribution of residual stress. However, these models are typically limited to specific materials, machining techniques, and experimental conditions, with their predictive accuracy heavily dependent on the quality of the data and their generalization capability. Consequently, the validation and calibration of empirical models are imperative for practical application. Common approaches in empirical modeling, such as regression analysis [23] and response surface methodology (RSM) [24], employ statistical techniques to establish empirical relationships between residual stress and various influencing factors (e.g., material properties, cutting parameters, tool parameters). These relationships are frequently represented by polynomial [25], exponential [26], or sinusoidal functions [27].

Saini et al. [28] developed a model to investigate the effect of cutting parameters on residual stress generated during the hard turning of AISI H11 tool steel using ceramic tools. Employing Response Surface Methodology with a Box–Behnken experimental design, they conducted machining trials, measured residual stress via X-ray diffraction, and performed analysis of variance (ANOVA) on the results. Their findings identified feed rate and depth of cut as the dominant factors affecting residual stress, whereas cutting speed and tool nose radius exhibited a comparatively minor influence. Jiang et al. [29] quantified the impact of machining parameters on residual stress in the milling of curved thin-walled components. By developing a geometric model for undeformed chip volume and a corresponding volumetric residual stress model, coupled with polynomial fitting (as expressed in Equations (1) and (2)), they demonstrated that increasing the tool radius and decreasing the depth of cut significantly reduced the maximum residual tensile stress. Their work concluded that tool radius and depth of cut were the most influential parameters, thereby providing a theoretical foundation for machining parameter optimization.

(1) $$UCV=F\left(f_{z},R,a_{p},a_{e},\right)$$

(2) $$\sigma =\alpha \cdot z\cdot n\cdot {UCV}^{\beta }$$

where n represents the tool rotational speed, z is the number of teeth, α and β are influence coefficients derived from fitting simulation data.

Jiang et al. [30] studied the effect of a novel coated carbide turning tool featuring a micro-groove design on the radial distribution of surface residual stress during the machining of high-strength alloy steel. This investigation integrated cutting experiments, theoretical analysis, and finite element simulation. A comparison of surface residual stress distributions generated in 40CrMnMo steel by this new tool and a conventional turning tool under the same cutting parameters revealed that the micro-groove design enhanced the surface integrity by increasing both the depth and the maximum magnitude of the residual compressive stress layer while reducing the residual tensile stress.

**Figure 2:**
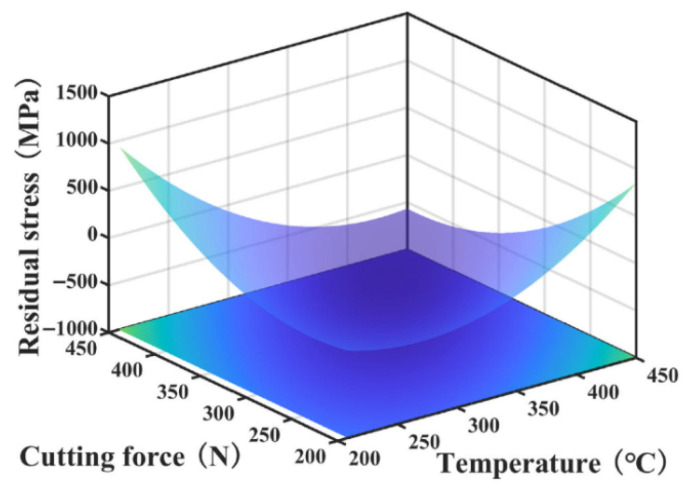
Relationship between cutting temperature, cutting force, and residual stress [32].

While prior research on machining-induced residual stress has primarily focused on qualitative assessments of mechanical and thermal loads, the quantitative separation of cutting force and heat effects remains a significant challenge, primarily due to the absence of a precise superposition mechanism. To address this, Jiang [31] developed a new empirical model for the superposition of residual stresses in milling. This model quantitatively analyzes the combined contribution of force-induced and heat-induced residual stresses, and its effectiveness was confirmed through both simulation and experimental validation. For the rough machining of Ti6Al4V, Li et al. [32] introduced a prediction method for residual stress based on cutting temperature and cutting force. Sensitivity analysis identified the friction coefficient, tool edge radius, and cutting speed as the dominant influencing factors (see Figure 2). Specifically, the friction coefficient and edge radius predominantly govern the thickness of the affected residual stress layer. In contrast, an increase in cutting speed tends to shift the residual stress profile towards a more tensile state. The established prediction model, which takes cutting temperature and cutting force as inputs, offers a novel approach for residual stress analysis by reducing the reliance on specific, predefined cutting conditions inherent in traditional models.

**Figure 3:**
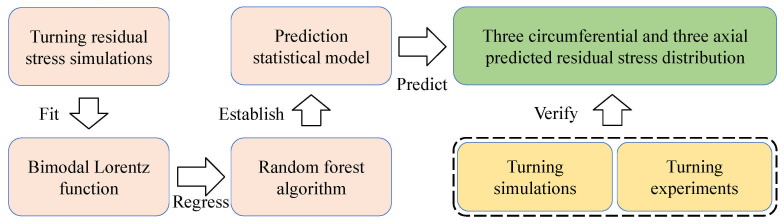
Main steps of the residual stress prediction process.

To leverage the strengths of empirical models and extend their applicability, machine learning techniques [33] have been introduced into the development of residual stress predictors. For instance, Peng et al. [34] proposed a prediction model based on a bimodal Lorentzian function, given in Equation (3). They utilized a Random Forest algorithm to establish a direct mapping between machining parameters and the coefficients of this function, enabling the prediction of the complete residual stress profile as a function of depth. The key steps of this prediction methodology are outlined in Figure 3.

(3) $$\sigma \left(h\right)=\sigma_{0}+\frac{2A_{1}}{\pi }\times \frac{0.2}{4h^{2}+0.04}+\frac{2A_{2}}{\pi }\times \frac{\omega }{4{\left(h-h_{c}\right)}^{2}+\omega^{2}}$$

where h is the independent variable (depth), $\sigma \left(h\right)$ is the dependent variable (residual stress), and there are five undetermined coefficients: $\sigma_{0}$, $A_{1}$, $A_{2}$, ω, and $h_{c}$.

Dong et al. [35] utilized a bimodal Gaussian function to model the residual stress profile, optimizing its coefficients via the Firefly Algorithm. This approach was validated against six independent turning tests, achieving a high fitting accuracy between 89% and 99.6%. Subsequently, Peng et al. [36] developed a semi-empirical model for predicting surface residual stress in turned Inconel 718, integrating data from physical experiments and finite element simulations. A key innovation of their work was the first-time inclusion of tool geometry parameters—specifically the cutting edge inclination angle, rake angle, and central cutting edge angle—into the predictive equations, offering novel theoretical insights for managing distortion in machined aerospace superalloy parts. Furthermore, based on foundational work regarding the fatigue behavior of titanium alloys [37], Lai et al. employed a response surface methodology to construct a multi-factor response model. This model successfully elucidates the mathematical relationships governing the effects of turning parameters on both surface roughness and surface residual stress.

(4) $$y=\gamma_{0}+\sum_{i=1}^{n}\gamma_{i}x_{i}+\sum_{i=1}^{n}\gamma_{i}x_{i}^{2}+\sum_{i=1}^{n}\sum_{j=1}^{n}\gamma_{ij}x_{i}x_{j}$$

where y denotes the response value, $\gamma_{0}$, $\gamma_{i}$, and $\gamma_{ij}$ are constants, $x_{i}$ and $x_{j}$ represent the independent variables for factors i and j, and n indicates the total number of parameters.

**Figure 4:**
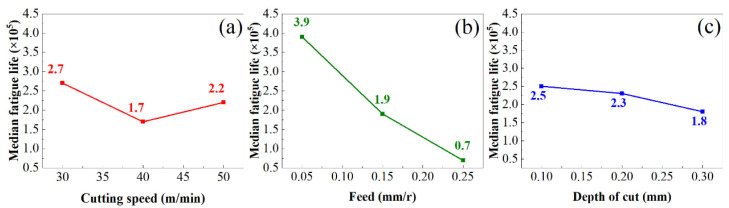
Influence of cutting parameters on residual stresses [38]. (**a**) Cutting speed; (**b**) Feed; (**c**) Depth of cut.

In a related study on TC17 titanium alloy, Wang et al. [38] investigated the effect of turning parameters on surface integrity and, consequently, fatigue life. Using an orthogonal experimental design coupled with bending fatigue tests, they identified a significant correlation between key surface integrity indicators and fatigue performance. They subsequently developed a mathematical model to quantify this relationship (Figure 4). Their principal findings indicated that feed rate exerts the most substantial influence on fatigue life, followed by depth of cut and then cutting speed. Additionally, the presence of surface residual compressive stress and elevated microhardness was confirmed to enhance the service life of components significantly.

**Figure 5:**
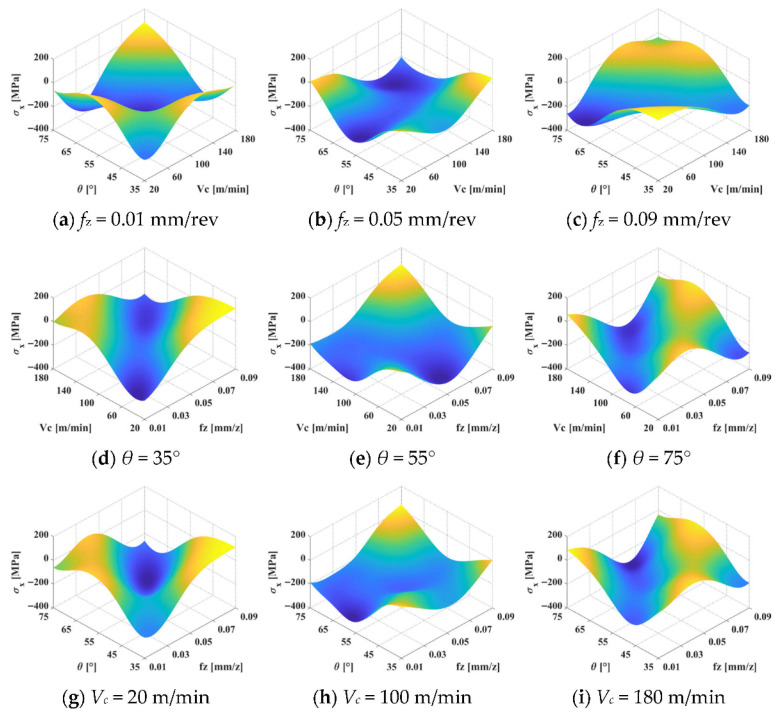
Interactive effects of cutting parameters on surface residual stress in the x-direction [39].

In the context of milling, Wang et al. [39] developed a Radial Basis Function (RBF) neural network model, calibrated with both simulation and experimental data, to predict surface residual stress during the multi-axis milling of titanium alloys. By constructing a three-dimensional numerical model, they analyzed the interactive effects of machining parameters on residual stress (Figure 5). This approach facilitates the identification of optimal processing conditions for controlling residual stress in Ti6Al4V workpieces under multi-axis milling operations. Based on end-milling experiments, Cheng et al. [40] introduced a surface residual stress prediction method that integrates physically measured cutting parameters with Gaussian Process Regression (GPR). Their methodology involves capturing cutting force and temperature signals, extracting relevant features from both time and frequency domains, and encoding the workpiece’s geometric characteristics. Key predictive features were selected using a Random Forest algorithm, with the circumferential cutting force identified as the most influential. By optimizing the GPR hyperparameters via grid search, they established a prediction model that demonstrated superior performance compared to conventional techniques. Wittich et al. [41] performed a comparative analysis of multiple polynomial regression, Takagi-Sugeno models, and Gaussian Process Regression for prediction tasks. They also proposed a model-based strategy for optimizing cutting parameters to concurrently meet requirements for low surface roughness and a favorable residual stress state. It is important to note that while machine learning methods often achieve superior predictive accuracy in residual stress estimation, they exhibit notable limitations regarding interpretability and robustness. In contrast to classical statistical–empirical models, machine learning techniques (e.g., random forests, neural networks, and Gaussian process regression) frequently operate through high-dimensional feature mappings whose internal parameters lack physically interpretable meaning. This “black-box” nature makes it challenging to elucidate the underlying mechanisms linking cutting parameters to the resulting residual stress. Moreover, the predictive performance of these data-driven models is highly dependent on and sensitive to the distribution of the training data. When process parameters, material types, or tool conditions deviate from the scope of the training dataset, their extrapolation capability and stability can degrade considerably. Traditional statistical–empirical models, such as multiple regression and response surface methodology, offer a counterpoint. Although their ability to capture complex nonlinear relationships is limited, their parameters have clear engineering significance and tend to yield more stable predictions, especially under small-sample conditions. Consequently, from a practical engineering standpoint, machine learning approaches are better suited for scenarios with abundant data and relatively stable operating conditions. Classical empirical models, however, retain distinct advantages in interpretability and robustness. This contrast underscores the need for and motivates the subsequent development of hybrid or physics-constrained modeling frameworks that aim to combine the strengths of both paradigms.

In conclusion, while existing empirical models, built upon localized experimental data, can capture residual stress profiles under specific test conditions, they suffer from limited generalizability. Their inability to adequately represent the complex coupling effects of multiple factors constrains their utility in practical engineering applications. Moreover, these models are vulnerable to measurement inaccuracies, often providing only qualitative trend indications, and fail to elucidate the fundamental mechanisms governing residual stress formation and evolution. Consequently, the development of advanced, multi-scale theoretical frameworks that couple multiple physical fields is essential for systematically unraveling and explaining the underlying generation mechanisms of machining-induced residual stress.

### 2.2. Analytical Models

**Figure 6:**
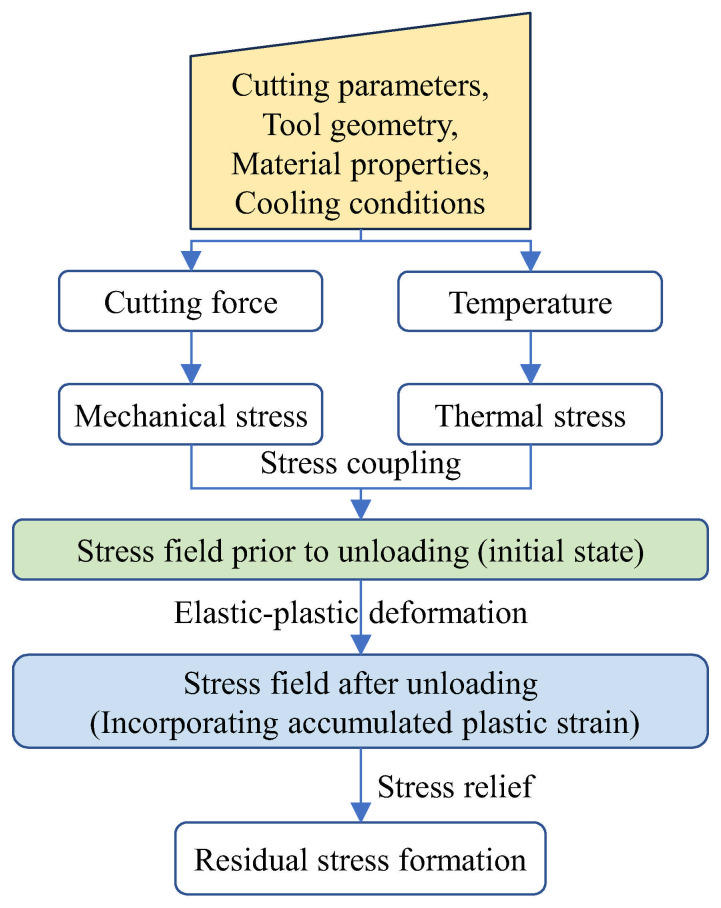
Brief flowchart for residual stress modeling using an analytical approach.

The foremost strengths of analytical models are their exceptional computational efficiency and their capacity to deliver clear physical insight into the intrinsic mechanisms governing residual stress formation [42]. These models work by establishing analytical frameworks to dissect the generation of mechanical and thermal stresses during machining, thereby offering a transparent view of the residual stress development process. A common simplification, however, is the assumption of two-dimensional orthogonal cutting conditions. A schematic outlining the typical analytical modeling workflow is presented in Figure 6.

**Figure 7:**
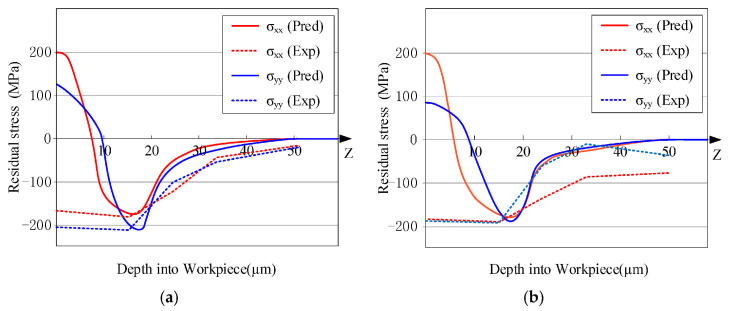
Predicted residual stress distribution in a machined surface [44]. (**a**) Case 1; (**b**) Case 2.

Su [43] developed an integrated analytical model that incorporates cutting forces, thermal effects, and material flow to predict residual stresses in orthogonal cutting, milling, and turning operations. Employing a layer-by-layer loading analysis to account for thermo-mechanical coupling, the study identified the friction coefficient as the most influential parameter on the predictions. The results also revealed discrepancies in predicting residual stress within the surface white layer, highlighting the need for future models to consider phase transformation effects. Yue et al. [44] proposed a predictive algorithm for milling-induced residual stress based on a plane stress assumption. The algorithm follows a procedural calculation of the loading and unloading stages (Figure 7), identifying the residual stress peak as the factor most critical to workpiece fatigue life. While the model achieved high accuracy in predicting near-surface stresses, significant errors were noted at greater depths. Furthermore, the model predicted that increased cutting speed, by intensifying thermal effects, elevates the proportion of surface tensile stress—a finding that aligns with the conclusions reported by Su [43].

**Figure 8:**
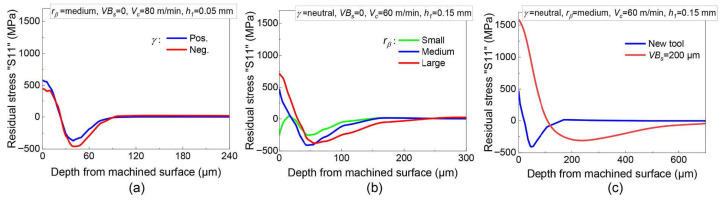
Influence of tool geometry on residual stresses distributions. Effect of (**a**) rake angle; (**b**) edge radius; and (**c**) flank wear [47].

Tool geometry also exerts a considerable influence on residual stress. Specifically, the plowing effect caused by the tool’s cutting edge radius alters the instantaneous stress field, which in turn affects the final residual stress state. Moreover, tool wear introduces additional mechanical loading. It acts as a secondary frictional heat source, generating a more complex transient stress field during cutting that significantly modifies the resultant residual stress distribution. To address these factors, Zhang et al. [45] presented an analytical model founded on an equivalent stress method that explicitly considers both tool nose radius and flank wear length. Liang et al. [46] further advanced this area by developing a multi-physics prediction model that incorporates tool wear. Their model accounts for the frictional forces and heat generated under flank wear conditions. Experimental validation confirmed the model’s effectiveness in characterizing residual stress distributions under varying tool wear states, enabling the establishment of a quantitative relationship between residual stress and tool wear condition. Liu et al. [47] employed the finite element method to analyze the effects of tool geometry on temperature, cutting forces, equivalent plastic strain, and residual stress (Figure 8). Their findings indicated that tools featuring a negative rake angle and a sharp edge radius typically induce higher stress on the machined surface than those with a positive rake angle. Additionally, increased flank wear was found to reduce local everyday stress by extending the flank contact length, thereby diminishing subsurface compressive stress. From a modeling standpoint, existing analytical models that account for tool wear can be systematically classified according to the method used to incorporate wear effects. The first category employs an equivalent geometric correction approach, which models the wear-induced increase in edge radius and alteration of the rake angle as modified tool-geometry parameters. These corrected parameters are then used to recalculate contact length, cutting forces, and thermal load distributions. Their simplicity and high computational efficiency characterize models in this category. However, their predictive accuracy is highly dependent on empirical calibration of wear-related geometric parameters and often fails to capture the dynamic progression of wear adequately. The second category accounts for wear by modifying parameters such as the friction coefficient and heat partition coefficient at the tool-workpiece interface. These models indirectly account for wear’s influence on residual stress by adjusting the interface friction conditions and heat source intensity. While capable of reflecting some thermal-mechanical load changes due to wear, their parameters are susceptible to specific operating conditions, limiting their generalizability. The third category is founded on a coupled thermo-mechanical framework. It treats wear as a comprehensive factor that simultaneously affects contact conditions, load distribution, and heat transfer pathways. This enables a more systematic description of how wear influences the underlying mechanisms of residual stress formation. The trade-off, however, is increased model complexity and a greater demand for input parameters.

In summary, analytical models based on equivalent geometry or friction correction are better suited for rapid trend analysis, whereas coupled thermo-mechanical analytical models offer superior physical consistency. The engineering application of the latter, nevertheless, remains challenging due to the difficulty in acquiring the necessary parameters. This comparison underscores that the chosen modeling approach critically governs the scope and generalizability of an analytical model for predicting residual stresses.

Beyond tool-related factors, the inherent properties and evolving microstructure of the workpiece material directly govern its mechanical response. Consequently, accurately predicting residual stress often requires integrating these material characteristics into stress field models to achieve higher fidelity. For instance, Pan [48] enhanced the traditional Johnson-Cook (J-C) constitutive model by incorporating a grain-size-dependent term to capture the influence of grain size on flow stress, yielding a modified J-C model (Equation (5)). This enhanced material model was then integrated into a standard residual stress prediction framework to create an analytical model for orthogonal turning. This model uniquely accounts for the effects of dynamic recrystallization and the associated grain growth during machining.

(5) $$\sigma =\left(A_{hp}+K_{hp}d^{-0.5}+B\bar{\varepsilon^{n}}\right)\left(1+Cln\frac{\dot{\bar{\varepsilon }}}{\dot{{\bar{\varepsilon }}_{0}}}\right)\left[1-{\left(\frac{T-T_{0}}{T_{m}-T_{0}}\right)}^{m}\right]$$

where d is the average grain size. Building upon this modified constitutive description, Pan et al. [49] introduced a method for predicting residual stress in the orthogonal turning of Ti6Al4V, utilizing a Mechanical Threshold Stress (MTS) model. Their approach refines the traditional J-C relation by integrating key microstructure-level variables such as dislocation interactions and grain boundary resistance, leading to an enhanced constitutive model given in Equation (6) that more accurately captures the material’s flow stress behavior. This improved model was subsequently integrated into an analytical framework for predicting residual stress. By performing coupled thermo-mechanical analysis to determine machining-induced loads and the thermal field, the model predicts the subsurface residual stress distribution. Validation studies indicated that the model successfully replicated the general trend of residual stress variation with depth. However, discrepancies were observed in the predicted stress magnitude at the immediate surface, attributed to experimental oxidation effects.

(6) $$\sigma =\sigma_{a}+\left[s_{i}\left(\dot{\varepsilon },T\right)\sigma_{i}+s_{s}\left(\dot{\varepsilon },T\right)\sigma_{s}+s_{D}\left(\dot{\varepsilon },T\right)\sigma_{D}\right]\frac{G}{G_{0}}$$

where $\sigma_{a}$ denotes the athermal stress component; $s_{i}$, $s_{s}$, $s_{D}$ are microstructure-sensitive deformation variables dependent on thermal kinematics, and strain rate $\dot{\varepsilon }$, $\sigma_{i}$, $\sigma_{s}$, $\sigma_{D}$ represent the resistances stemming from dislocations and solute atoms; and G is the temperature-dependent shear modulus.

The aforementioned analytical models for residual stress prediction are primarily based on the two-dimensional theory of orthogonal turning. However, the intermittent and cyclic nature of milling—a far more prevalent industrial process—renders these orthogonal-cutting-based theories insufficient for direct application. Consequently, scholars have pursued specific research on predicting milling-induced residual stress. A common strategy involves leveraging orthogonal cutting theory as a foundation: the milling cutting edge is discretized into infinitesimal elements, each treated as an oblique cutting segment. Cutting force analysis is then conducted on an equivalent plane using an orthogonal cutting model. For temperature prediction, the same discretization principle is applied; the temperature rise caused by each cutting element within a given time increment is calculated via coordinate transformation and a moving plane heat source technique. The corresponding stress generated by the element is computed, and the final residual stress profile for the milling process is determined by synthesizing the effects of loading, unloading, and stress relaxation across all elements and time steps [50,51]. Moving beyond standard milling, Huang et al. [52] developed a three-dimensional analytical model for residual stress in deep-hole drilling based on eigenstrain theory. This model simplifies the complex cutting process by applying equivalent mechanical loads, incorporates a guide pad contact model based on Hertzian contact theory, performs incremental plastic analysis using a modified McDowell algorithm, and efficiently reconstructs the residual stress field using eigenstrain theory. Cai et al. [53] proposed an analytical model for end-milling residual stress that incorporates boundary layer lubrication effects and an orthogonal cutting-based chip formation model. Their method first reduces the 3D milling process to a 2D orthogonal cutting equivalent to compute lubrication-influenced cutting forces and temperature, then employs elastoplastic theory to calculate the resultant residual stress. Yao et al. [54] presented a prediction method based on microhardness measurement, formulating a mathematical model for equibiaxial stress indentation that simplifies the acquisition of parameters. However, it requires substantial experimental data for calibration. Furthermore, emerging research attention is being directed towards the effect of initial stress states in multi-pass machining [55]. Findings suggest that residual stress accumulates with each successive layer of material removal, highlighting the necessity for predictive models to incorporate a mechanism for superimposing stresses from previous machining steps.

Additionally, efforts have been made to refine traditional stress-relaxation methodologies. Huang et al. [56] introduced a novel relaxation-free analytical approach grounded in inclusion theory, which circumvents the conventional stress relaxation step altogether. Validation experiments demonstrated that the predictions from this method were in close agreement with those obtained from traditional relaxation-based techniques and also exhibited strong correlation with both experimental measurements and established numerical simulation data. Compared to conventional methods, this relaxation-free analytical approach maintains the high computational efficiency characteristic of analytical models while offering a more streamlined solution procedure and providing clearer insights into the fundamental mechanisms of residual stress generation.

Shan et al. [57] formulated the cutting force model by integrating Oxley’s predictive theory, the J-C constitutive model [58], and slip-line field theory. The cutting temperature model was developed based on the author’s earlier research [59], incorporating elements from the Huang-Liang model [60] and the Komanduri-Hou model [61,62,63]. Utilizing inclusion theory in conjunction with a shear lag model [64], the distributions of mechanical and thermal stresses were succinctly presented. For the machining of 304 austenitic stainless steel, Zhang et al. [65] constructed a multi-physics coupled analytical model. This framework employs orthogonal cutting theory to predict cutting forces and the distributions of stress, strain, and temperature. It further simulates microstructural evolution through strain-induced martensitic transformation kinetics, predicts changes in microhardness by coupling dislocation density evolution with phase transformation effects, and finally determines the residual stress state via a mechanical relaxation process. The model’s outputs for cutting force, martensite content, microhardness, and residual stress profile all showed excellent agreement with experimental measurements. Other notable contributions include Ji et al. [66] creating a physics-based analytical model that accounts for thermo-mechanical coupling to predict residual stress under Minimum Quantity Lubrication (MQL) conditions; Wang et al. [67] deriving the orthogonal cutting stress field using the radial return method and introducing an R-R approach to mitigate issues of stress discontinuity and yield surface drift inherent in traditional methods, subsequently improving temperature field modeling by integrating shear, plowing, and friction effects to achieve a prediction error below 12%; Yang et al. [68] developing an analytical model that considers the size effect of the tool nose radius on machining-induced residual stress to better elucidate its influence mechanism for field optimization; and Huang et al. [69] implementing incremental plastic strain theory to facilitate the real-time tracking of dynamic cutting stress waves.

To summarize, analytical models have successfully enabled the efficient prediction of turning-induced residual stress through their mechanism-driven foundations. Nonetheless, significant challenges remain, particularly in handling intricate multi-field couplings and adapting to the complexities of real-world industrial machining scenarios. Future research directions should focus on integrating real-time process monitoring data, developing algorithms for adaptive model updating, and extending the applicability of these models to novel manufacturing contexts such as additive manufacturing. It is important to recognize that the pursuit of analytical tractability necessitates simplifications in tool geometry, interfacial friction, and material constitutive behavior [70,71]. Predictions of residual stress are profoundly sensitive to these underlying assumptions; consequently, a critical understanding of the limitations associated with each modeling technique is paramount.

### 2.3. Finite Element Models

When the simplifying assumptions inherent in analytical models become insufficient to capture the complexities of actual machining phenomena, the FEM emerges as a powerful alternative. As a robust numerical computation technique, FEM works by discretizing both the workpiece and the cutting tool into a finite collection of interconnected elements. It then simulates the continuous evolution of key physical fields throughout the cutting process by solving the underlying system of governing partial differential equations [72,73]. By numerically recreating the coupled thermomechanical effects of cutting, FEM is one of the most precise approaches currently employed for predicting residual stress. The method’s effectiveness hinges on constructing high-fidelity physical models and configuring them with appropriate material constitutive laws, accurate boundary conditions, and stable solution algorithms. This setup facilitates a dynamic analysis of the evolving stress field. Ultimately, through the development of integrated mechanical and thermal models of the process, FEM enables the prediction of residual stress distributions on machined surfaces. This capability provides rapid numerical estimates of residual stress, offering significant savings in time and experimental expenditure [74].

**Figure 9:**
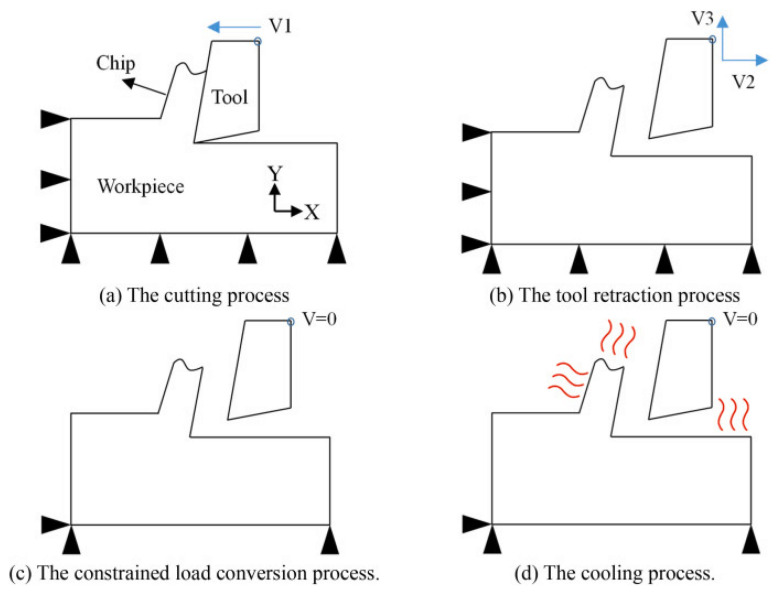
The four stages of predicting residual stress using a finite element model [75].

The practical application of FEM for residual stress prediction entails building a detailed simulation model of the machining operation within specialized finite element software. The simulation sequentially replicates critical real-world stages—including material removal (cutting), tool retraction, and subsequent workpiece cooling—to computationally derive the final residual stress profile imparted to the surface. A typical representation of this simulation workflow, comprising four key stages, is shown in Figure 9 [75].

Mesh generation constitutes a foundational step in Finite Element Analysis, with a direct bearing on both the accuracy of the simulation results and the efficiency of the computation. The characteristics of the mesh have a profound impact on numerical precision and the required computational time. Four main meshing strategies are commonly employed: the Updated Lagrangian [76,77], the Arbitrary Lagrangian–Eulerian (ALE) [78], the Pure Lagrangian [79,80], and the Coupled Eulerian–Lagrangian (CEL) [81] methods. A comparative summary of their underlying principles and respective advantages is provided in Table 1. Given that metal cutting processes involve significant material deformation and strain, the ALE method has found widespread application in this context. Its primary advantage lies in its ability to handle significant deformation problems more effectively than other methods by substantially reducing issues related to mesh distortion and numerical instability.

During the initial phase of applying the FEM to residual stress prediction, research efforts were primarily focused on analyzing how macroscopic factors, including material constitutive parameters and chip formation characteristics, influence residual stress distribution. For instance, Jiang et al. [82] examined the influence of undeformed chip thickness (UCT) on residual stress, suggesting strategies for optimizing machining parameters to control its distribution and demonstrating predictions for residual tangential and radial stresses in various coordinate systems. Umbrello et al. [83] explored the sensitivity of predictions for cutting force, chip morphology, temperature distribution, and residual stress to five different sets of J-C constitutive parameters, validating their finite element model through comparison with experimental data. Shi et al. [84] developed a finite element model to predict residual stresses on pre-stressed dry-grinding surfaces by incorporating the coupled effects of the thermal field, pre-stress, and phase transformation. Sahu et al. [85] developed a predictive model for residual stress in the turning of the titanium alloy Ti6Al4V by integrating 3D finite element simulation, response surface methodology, and X-ray diffraction experiments, and subsequently optimized the cutting parameters. By employing a finite element model integrated with slip-line theory, Liu et al. [86] analyzed the surface residual stress generated during cryogenic turning of Ti6Al4V. This model accounted for both mechanical and thermal stresses, and its validity was confirmed experimentally.

Subsequent iterations and enhancements in finite element simulation capabilities, fueled by technological breakthroughs like user-defined subroutines and the customizable features of commercial software, have substantially deepened the scope of inquiry. In recent years, the research focus has expanded to encompass material microstructural evolution during machining. This includes investigating the dynamic response of features like grain size and dislocation density, understanding coupling mechanisms with pre-existing stress states, and accounting for work-hardening effects factors that introduce greater complexity but better approximate real machining conditions [87,88,89]. This shift is driving the gradual development of a predictive modeling framework for residual stress that integrates multi-scale characteristics. The research objective has also evolved beyond merely capturing the general trend of residual stress distribution; there is now a concerted effort to achieve higher predictive accuracy and broader model applicability. Achieving this requires a deeper integration of multiphysics couplings and the inclusion of finer process details into the models, thereby addressing the oversimplifications inherent in earlier approaches. Notable progress has been made, especially in refining the representation of material constitutive behavior. This involves developing or adopting more sophisticated constitutive models, such as those accounting for dynamic strain aging, physics-based models that track the evolution of dislocation density, and models incorporating transformation plasticity for steels undergoing phase changes during thermal cycles [90]. These advanced formulations enable a more accurate capture of material thermomechanical responses under extreme machining conditions, resulting in improved precision in predicting residual stress profiles.

**Figure 10:**
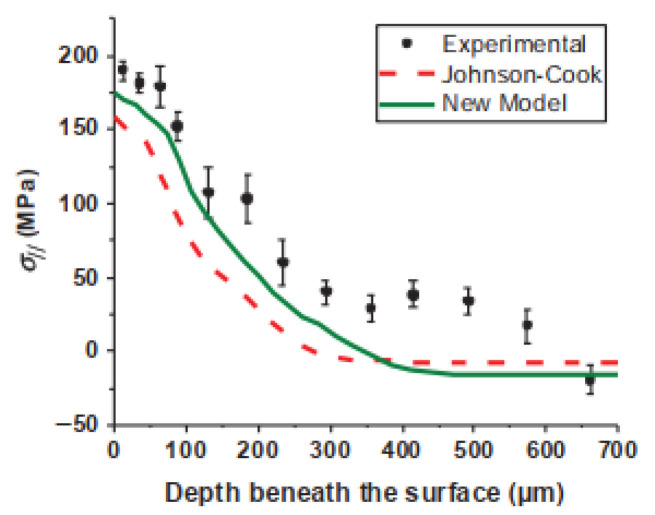
Comparison of experimental and predicted residual stresses in the cutting direction [91].

A specific example is the work of Denguir et al. [91], who introduced a new constitutive model for Oxygen-Free High-Conductivity (OFHC) copper, given in Equation (7). This model holistically considers the combined effects of stress state and microstructure on flow stress. When implemented within an orthogonal cutting finite element simulation, it demonstrated a marked improvement in predicting surface integrity metrics compared to the standard J-C model, as shown in Figure 10.

(7) $$\bar{\sigma }=\left[A+B\varepsilon^{n}\right]\times \left[1+C\ln\left(\frac{\dot{\varepsilon }}{\dot{\varepsilon_{0}}}\right)\right]\times \left[1-{\left(\frac{T-T_{\mathrm{room}}}{T_{m}-T_{\mathrm{room}}}\right)}^{m}\right]\times H\left(\varepsilon ,\dot{\varepsilon },T\right)\cdot \left[1-c_{\eta }\left(\eta -\eta_{0}\right)\right]$$

In this constitutive equation, the individual terms correspond to distinct physical mechanisms: the first term captures strain hardening, the second represents the strain rate effect, the third accounts for thermal softening, the fourth models microstructural (or phase) transformation effects, and the fifth introduces the influence of stress state, specifically via the parameter η, defined as the ratio of hydrostatic stress to the Von Mises equivalent stress.

Ding et al. [92] formulated a flow stress model that incorporates phase transformation effects by integrating a standard constitutive framework with a transformation plasticity model. This led to the development of a coupled thermo-mechanical-phase transformation model for simulating single abrasive grit micro-grinding, expressed in Equation (8). The model was validated through experiments, including micro-hole drilling and surface grinding tests under varied process parameters, which helped elucidate how phase transformations affect residual stress. Key findings show that phase transformation substantially reduces residual tensile stress in the tangential direction beneath the surface. The maximum residual compressive stress was located on the axial (top) surface, whereas the maximum tensile stress occurred in the tangential subsurface region. This work provides the first quantitative analysis of the dynamic impact of transformation plasticity on residual stress, offering valuable theoretical insights for manufacturing high-precision components.

(8) $$\sigma_{k}=\left[A+B{\left(\varepsilon^{cp}+\varepsilon_{k}^{\left(tp\right)}\right)}^{n}\right]\left(1+Cln{\frac{\dot{\varepsilon }}{\dot{\varepsilon_{0}}}}^{*}\right)\left[1-{\left(T^{*}\right)}^{h}\right]$$

where $\varepsilon^{cp}$ is the macroscopic equivalent plastic strain, $\varepsilon_{k}^{\left(tp\right)}$ is the equivalent plastic strain associated with the k-th phase transformation step, ε is the effective plastic strain rate, $\dot{\varepsilon_{0}}$ is the reference strain rate, A is the initial yield strength, B is the hardening constant, n is the hardening exponent, C is the strain rate sensitivity constant, and h is the thermal softening exponent.

A review of the existing literature on finite element-based residual stress prediction indicates that research employing this method is predominantly dedicated to simulating the machining process under realistic operating conditions, a focus that constitutes a significant thrust in the field. By explicitly incorporating key influencing factors present in actual machining, the finite element approach is capable of predicting both the magnitude and the spatial distribution of residual stress on workpiece surfaces with considerable accuracy. A significant limitation, however, is the method’s characteristically low computational efficiency. This constraint severely restricts the achievable complexity and reliability of the models that can be constructed. The issue of computational expense is seldom addressed directly, resulting in a predominance of studies based on two-dimensional models. In contrast, three-dimensional cutting simulations, which involve a steep, often exponential, increase in computational demand, remain relatively uncommon in the literature due to impractical solution times. Although FEM can explicitly incorporate real-world machining conditions—including complex tool geometry, realistic cutting paths, multi-pass operations, and coupled thermo-mechanical phenomena—this high physical fidelity is generally achieved at substantial computational cost. In contrast, hybrid models aim to reproduce many of these real-world effects in an approximate but computationally efficient manner. By replacing detailed chip–tool–workpiece interactions with equivalent mechanical and thermal loads, reduced-order representations, or physics-informed surrogate models, hybrid approaches can capture the dominant influences of realistic machining conditions on residual stress evolution while significantly reducing simulation time. As a result, hybrid models often provide predictive trends comparable to full FEM simulations for surface and subsurface residual stress distributions, particularly in terms of stress sign, peak magnitude, and depth-wise variation, at orders of magnitude lower computational cost. This comparison highlights that hybrid models serve not as direct replacements for FEM, but as efficient alternatives when repeated simulations, process optimization, or large-scale parametric studies under realistic machining conditions are required.

### 2.4. Hybrid Model

While the FEM offers a physics-based simulation of the cutting process, it is hampered by significant challenges, including severe mesh distortion, complexities in defining tool-workpiece contact, and prohibitively long computation times for large-scale analyses. These issues are particularly pronounced and remain inadequately resolved for simulations involving multi-pass operations and three-dimensional turning scenarios [93]. On the other hand, purely data-driven neural network models provide rapid computation but frequently lack integration of physical laws, rendering their predictive reliability and generalization capacity highly dependent on the scope and quality of the training data [94]. In response to these limitations, researchers have turned to hybrid modeling strategies. These approaches aim to synergize the advantages of two or more distinct predictive methodologies to mitigate the shortcomings inherent in any single method. Examples include merging the mechanistic understanding provided by analytical models with the adaptability of empirical models, or employing high-fidelity data generated from detailed FEM simulations to train efficient surrogate models that retain predictive accuracy at a fraction of the computational cost.

**Figure 11:**
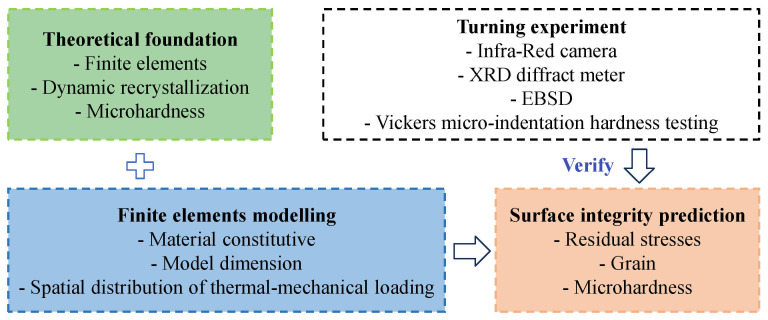
Hybrid modeling strategy for the turning process.

During the formative phase of hybrid model research, a pivotal concept emerged to sidestep the computational burden of directly modeling the intricate interactions among the tool, chip, and workpiece. This involved representing the tool’s mechanical and thermal action through “equivalent loads,” which are then applied as boundary conditions to a simplified finite element model of the workpiece alone, enabling the subsequent calculation of residual stress [95]. Illustrating this approach, Wang et al. [96] introduced an equivalent load method utilizing a multi-scale FEM framework. This model predicts the evolution of residual stress from a single microscopic cut to macroscopic multi-pass machining, accounting for the effects of tool geometry and processing parameters. Ren et al. [97] employed a coupled thermo-mechanical simulation to forecast both residual stress and microstructural alterations on a machined surface. By analyzing the numerical results, the separate contributions of thermal and mechanical loads were discerned, and the influence of cutting parameters on residual stress and microstructure was elucidated. The study culminated in the proposal of a specific hybrid modeling strategy, depicted in Figure 11.

**Figure 12:**
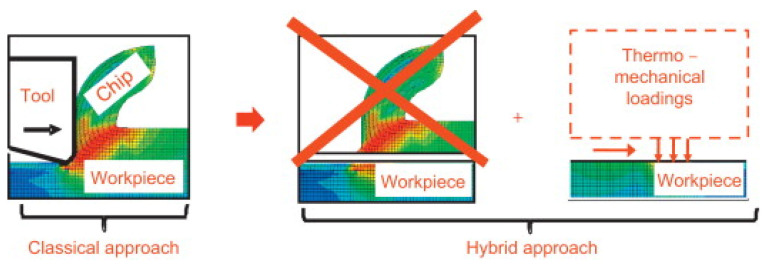
Principle of the model [98].

**Figure 13:**
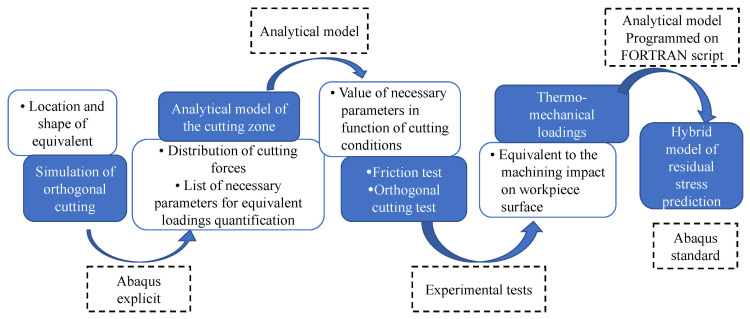
Methodology of the hybrid approach for predicting residual stress.

To advance the use of three-dimensional models in FEM-based residual stress prediction while addressing the associated computational expense, Valiorgue et al. [98] implemented the equivalent load methodology for predicting residual stress in the finish machining of 304 L stainless steel. This work led to the development of a generalized three-dimensional predictive model, with its underlying principle and the associated residual stress evolution process illustrated in Figure 12. Pushing the concept further, Rami et al. [99] proposed a more sophisticated distribution of equivalent thermo-mechanical loads to predict surface residual stress in turned 4140 steel. Departing from prior models that assumed a semi-infinite workpiece, their approach applied the equivalent loads to a finite one-eighth cylindrical segment of the workpiece, representing a refinement of the hybrid modeling technique. The corresponding workflow for this method is presented in Figure 13.

In addition to forward prediction of residual stress distributions, hybrid models are increasingly employed for parameter identification and inverse calibration. In such applications, experimentally measurable quantities—such as cutting forces, cutting temperatures, or surface residual stress profiles—are used as reference targets to identify effective thermo-mechanical loads, friction coefficients, or material-related parameters within analytical or finite element frameworks. By coupling physics-based models with data-driven optimization or learning algorithms, hybrid approaches enable the systematic adjustment of model parameters to improve agreement with experimental observations. This capability significantly enhances model robustness and transferability across different machining conditions, materials, and tool states. It is particularly valuable for industrial applications where direct measurement of internal model parameters is impractical.

**Figure 14:**
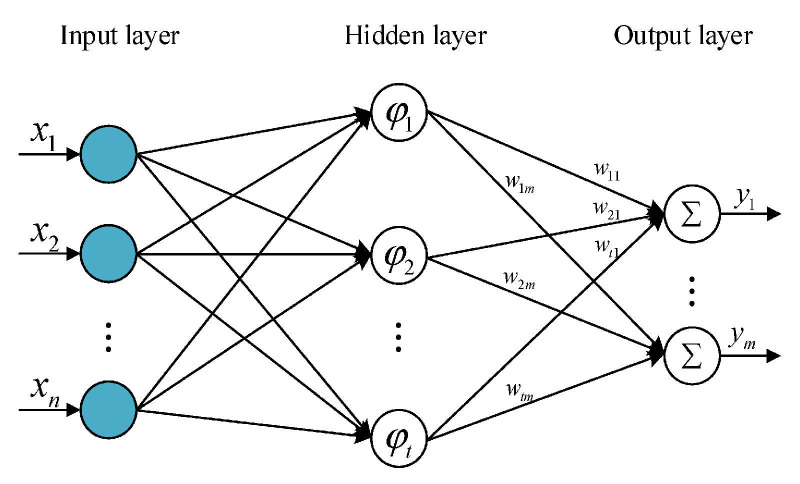
RBF neural network structure [39].

Driven by the rapid advancement of artificial intelligence and digital twin technologies, research into hybrid models has entered a distinct new phase. Here, physics-informed machine learning models and digital twins have emerged as prominent research directions in frontier physics. Umbrello et al. [100] introduced an artificial neural network to predict subsurface residual stress in turning operations and to inversely identify the cutting parameters required to achieve a specific stress state. The model was trained on data generated from finite element simulations and validated against experimental results from the literature. It demonstrated robust performance in both forward prediction and inverse design, with reported prediction errors ranging between 4% and 10%. Wang et al. [39] developed a radial basis function (RBF) neural network model based on simulation and experimental data to predict surface residual stress during multi-axis milling of Ti6Al4V. They observed a strong correlation between the experimental and predicted results. This is shown in Figure 14.

A more recent and influential hybrid paradigm that has evolved is the FEM+ Artificial Neural Network (ANN) approach. Its fundamental principle involves using computationally intensive but high-fidelity FEM simulations to generate a comprehensive dataset mapping various cutting parameters to their corresponding residual stress outcomes. This numerically generated, high-quality dataset is then employed to train an ANN. Once trained, the ANN serves as an ultra-fast surrogate model, capable of delivering millisecond-level predictions of residual stress for new sets of cutting parameters, thereby overcoming the primary efficiency bottleneck of direct FEM simulation. Ambrogio et al. [101] were among the first to articulate this concept, highlighting that such a hybrid framework could facilitate not only forward prediction but also the inverse optimization of process parameters to attain a desired residual stress profile. Similarly, Lin et al. [102] used FEM to simulate chip formation and generate residual stress data, subsequently leveraging an ANN to learn the complex mapping from input parameters to stress output. This methodology represented a significant step in transcending the limitations of standalone models, paving the way for multidisciplinary integrated solutions.

**Figure 15:**
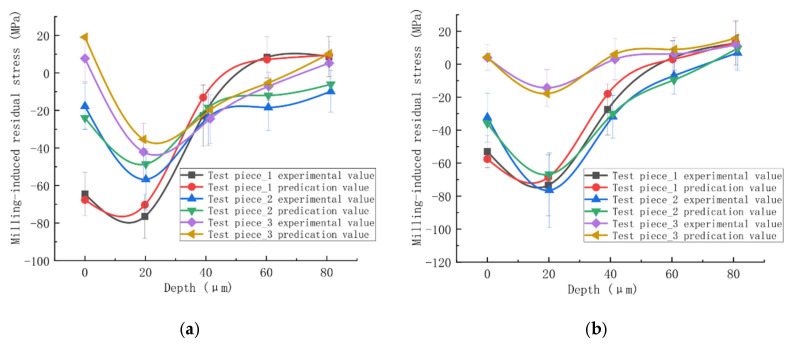
Prediction errors: (a) the end-milling; (b) the side-milling [106].

In the context of predicting residual stress induced by Laser Shock Peening (LSP), Bock et al. [103] implemented an innovative variant. A fast but approximate semi-analytical model was used as the base predictor. High-fidelity FEM simulations provided the reference data to train an ANN specifically to learn the systematic deviation or error between the semi-analytical model’s predictions and the more accurate FEM results. During application, predictions are made rapidly using the semi-analytical model and then refined by the corrections provided by the trained ANN, achieving accuracy comparable to that of detailed FEM at a fraction of the computational cost [104]. This approach, by explicitly embedding a physical (semi-analytical) model, offers greater robustness than black-box ANNs trained purely on experimental data. Other notable contributions include the work of Ji et al. [105], who proposed a hybrid neural network model for Minimum Quantity Lubrication (MQL) turning. They employed a combination of Simulated Annealing (SA) and the Levenberg–Marquardt algorithm for network training. They utilized a Genetic Algorithm (GA) to optimize cutting conditions aimed at minimizing surface tensile stress. Yi et al. [106] developed a prediction model integrating a Backpropagation (BP) neural network with a genetic algorithm. Through orthogonal experiment-based analysis of milling parameters, they observed a characteristic “spoon-shaped” residual stress profile along the depth direction. The prediction errors of their established models for both end-milling and side-milling operations were minimal, as visually confirmed in Figure 15.

Mahdi et al. [107] extended the application of hybrid modeling to the face milling of Al-6061-T3. Their orthogonal experiments revealed that a combination of high cutting speed, medium feed rate, and considerable depth of cut significantly elevated residual stress levels. They developed an ANN-based predictive model, introducing the innovative step of directly incorporating experimental data into the network’s error function, which facilitated swift optimization of machining parameters. Concurrently, Zhang’s research team [108] employed a Backpropagation neural network to forecast surface wear resistance in high-speed milling operations. The field has since evolved, with researchers no longer content with using FEM and ANN as merely separate, serially connected components. Instead, the current trend seeks a more fundamental integration, embedding the governing physical laws directly into the neural network’s learning framework—an approach exemplified by Physics-Informed Neural Networks (PINNs) and related variants. This generation of hybrid models is characterized by intelligent learning that is intrinsically guided and constrained by physical principles. This advancement allows hybrid models to tackle increasingly complex physical phenomena. For instance, when predicting residual stress in turning operations involving phase-sensitive materials, such as Ti6Al4V, a hybrid algorithm was proposed that accounts for microstructural evolution [109]. This model goes beyond simulating the macroscopic thermo-mechanical fields; it integrates the Johnson–Mehl–Avrami–Kolmogorov model to describe dynamic recrystallization, thereby directly correlating the predicted residual stress with the material’s internal microstructural changes and achieving substantially higher fidelity. Further innovations include the work of Zou et al. [75], who introduced a hybrid modeling method for predicting residual stress in Ti6Al4V turning that explicitly considers frictional contact. By combining finite element analysis with analytical techniques, they developed a novel model with a variable friction coefficient. Implementing this via secondary development in ABAQUS using the VFRIC interface led to a marked improvement in prediction accuracy. Elsheikh et al. [110] achieved superior accuracy compared to conventional ANNs by using an AI model whose parameters were fine-tuned with a Pigeon-Inspired Optimization algorithm for residual stress prediction in turning. Demonstrating the power of synergy, Zhou et al. [111] proposed a tri-hybrid model that seamlessly combines FEM, analytical analysis, and neural networks. These frontier studies collectively signal a clear trajectory towards more intelligent and sophisticated hybrid modeling systems.

To summarize, research specifically focused on “equivalent load”-based hybrid models remains limited and has predominantly addressed turning processes. The applicability of this theory is further constrained by the more complex load conditions inherent in milling, which stem from its intermittent cutting action and periodic loading patterns. Consequently, the general adaptability and broader acceptance of the “equivalent load” concept warrant further scrutiny. It is acknowledged, however, that hybrid models have demonstrably enhanced both the efficiency and accuracy of residual stress prediction. From a computational standpoint, they have rendered the use of three-dimensional finite element models for predicting machined surface residual stress a feasible endeavor in terms of time investment. Pending the wider adoption and validation of such integrated frameworks, the strategic combination of intelligent algorithms, finite element methods, and analytical models itself constitutes a practical hybrid approach for boosting predictive efficiency. A comparative summary of the strengths and weaknesses of the four primary model types discussed is presented in Table 2.

Although the four categories of residual stress prediction models differ fundamentally in their formulation and underlying assumptions, their practical value ultimately depends on how sensitively and accurately they can reproduce experimentally measured residual stress fields under realistic machining conditions. To enable an objective and application-oriented comparison in this regard, the prediction accuracy, computational cost, parameter sensitivity, scalability, and ease of application of representative models are systematically summarized in Table 3. Looking ahead, key challenges involve achieving a more profound fusion of physical principles with data-driven techniques—for example, by directly embedding partial differential equations, such as the heat conduction equation, into neural network architectures—and unraveling the coupling mechanisms between microstructural evolution at the micro-scale and the resulting macroscopic stress fields in cross-scale modeling endeavors.

## 3. Conclusions and Prospects

This paper has provided a systematic review and critical commentary on recent advancements, both domestic and international. A unified quantitative comparison of the four major model families is provided in Table 4 to facilitate objective assessment and model selection. In predictive modeling for machining-induced surface residual stress. Following a comparative analysis of the distinct features of various modeling methodologies and in light of current research trends and practical industrial needs, the limitations inherent to each model type and prospective future directions are synthesized below.

### 3.1. Empirical Models

These models are fundamentally dependent on fitting to substantial volumes of experimental data. While capable of providing predictions within narrowly defined conditions, they incur high development costs and exhibit limited portability and generalizability across different materials or process regimes. Future efforts should prioritize: (a) the creation of more efficient and economical experimental design and data fitting strategies to lower the barrier to model development; (b) the establishment of generalized empirical frameworks capable of accommodating wide ranges of process parameters and diverse material systems, thereby boosting their utility in practical engineering applications.

### 3.2. Analytical Models

Grounded in metal cutting theory and elasto-plastic mechanics, analytical models offer the advantages of high computational speed and transparent physical interpretation, facilitating the swift estimation of residual stress. Their primary drawback stems from the numerous simplifying assumptions typically required to achieve analytical tractability, which compromises prediction accuracy and restricts adaptability to complex, real-world machining scenarios. Significant improvements in model fidelity could be realized by focusing on the following: (a) The development of fully three-dimensional analytical formulations that more accurately capture the actual machining geometry and physical interactions, incorporating coupled thermo-mechanical and other relevant stress sources. (b) Advancing beyond the predominantly two-dimensional theories currently used for stress relaxation; novel methodologies for handling stress release and appropriate boundary conditions in 3D analytical models are needed. (c) Enhanced treatment of tool wear. Wear critically modifies cutting forces and thermal fields, profoundly affecting the outcomes of residual stress. Although recognized as important, detailed analytical modeling of specific wear mechanisms (e.g., crater wear, flank wear/pitting) remains underdeveloped. Given the inherent complexity of wear evolution, a promising path forward involves hybrid strategies that couple analytical models with finite element simulations to quantitatively describe wear progression and integrate its effects into the residual stress prediction chain.

### 3.3. Finite Element Models

FE simulations provide a high-fidelity representation of complex cutting physics, yielding residual stress predictions that closely approximate reality. Their most significant limitation is computational expense, particularly the disproportionately long time required to simulate the stress redistribution phase during workpiece cooling compared to the cutting phase itself. This severely hampers overall prediction efficiency. A crucial research thrust for enhancing the practical viability of the FE method, therefore, lies in devising efficient algorithms or reduced-order models specifically for the cooling stage to significantly reduce its computational overhead.

### 3.4. Hybrid Models

The hybrid paradigm, particularly concepts based on “equivalent loads,” applies mechanically and thermally representative loads (derived from analytical or empirical sources) to a finite element model of the workpiece. This strategy retains the strengths of detailed FE analysis while dramatically cutting computation time, presenting considerable potential. Present applications are primarily confined to turning operations, and model accuracy is highly sensitive to the fidelity of the equivalent load representation. Future research should concentrate on (a) strengthening the theoretical foundation and experimental validation of equivalent load methodologies; (b) extending their application to other processes like milling and grinding, which involve more complex, intermittent loading; (c) developing robust, efficient techniques for determining accurate equivalent loads. Moreover, a highly promising avenue is the integration of intelligent data-driven algorithms (e.g., machine learning) with physics-based methods (analytical or FE). Analyzing simulation or experimental data through these algorithms to build surrogate models or correction modules, which are then fused within a hybrid analytical-FE framework, constitutes a powerful pathway towards achieving both high efficiency and high accuracy in prediction.

In conclusion, the field of residual stress prediction is clearly advancing towards models that are more accurate, efficient, and broadly applicable. The path forward requires transcending the traditional boundaries that separate different modeling approaches. By fostering the complementary integration of multi-physics and multi-scale modeling techniques and leveraging the power of digitalization and artificial intelligence, the ultimate objective is to create robust, reliable predictive tools capable of handling complex industrial machining scenarios. Such tools would offer indispensable theoretical support for optimizing manufacturing processes and precisely controlling the in-service performance of engineered components.

## Figures and Tables

**Table 1:** Comparison of Finite Element Meshing Methods.

Method	Principle	Advantage	Disadvantage	Accuracy	References
Update Lagrangian	Remap the slightly distorted mesh to the new mesh	Efficient and adequate experimental validation	Neglecting microstructural evolution	Consistent trends, error 10%	[77]
ALE	Adaptive partitioning for large deformation problems	Balancing efficiency and precision	Too much model simplification	Mean error 14.28 Mpa	[78]
Pure Lagrangian	The mesh adheres to the material and deforms with its movement	Precise tracking of material deformation and microstructure evolution	Frequent re-meshing	Larger error, ALE closer to experimental value	[79]
CEL	Analyzed by thermal-mechanical coupling	Avoid mesh distortion, suitable for thermal-force coupling	Failure to consider micro-mechanisms	Maximum deviation 17.96%	[81]

**Table 2:** Advantages and disadvantages of prediction models.

Method	Advantage	Disadvantage
Empirical Model	(1) Simple and fast modeling, without the complex theoretical derivation (2) Less parameter requirements, only basic cutting parameters are needed	(1) Low accuracy, ignoring physical mechanisms (2) Depend on a large amount of experimental data (3) Unable to explain the mechanism
Analytical Model	(1) Clear mechanism (2) High computational efficiency (3) Convenient parameter sensitivity analysis	(1) Simplified assumptions are many (2) Poor interchangeability (3) Accuracy limited by theory
FEM	(1) High accuracy, can simulate complex physical fields (2) Wide applicability (3) Strong visualization	(1) High computational costs (2) Complex modeling and fine mesh required (3) Time-consuming simulation of cooling phase
Hybrid Model	(1) Balance efficiency and accuracy (2) High data utilization rate (3) High scalability	(1) Difficult to develop (2) Long validation period

**Table 3:** Features of prediction models.

Model Category	Accuracy vs. Experiments	Computational Cost	Sensitivity to Parameters	Ease of Application	References
Empirical	±15–30% (within data range)	Very low (seconds)	High sensitivity to data distribution	High	[28,112]
Empirical + ML	±10–20% (training domain)	Low–medium	Sensitive to training data & hyperparameters	Medium	[41,113,114]
Analytical	±10–25% (simplified cases)	Very low	Sensitive to assumptions & friction/heat partition	Medium	[10,115,116]
FEM (macro-scale)	±5–15%	High (hours–days)	Sensitive to mesh, material & contact models	Low	[72,117]
FEM (microstructure-aware)	±3–10%	Very high	Highly sensitive to material models	Very low	[42,118]
Hybrid	±5–12%	Medium	Moderate (physics-constrained)	Medium–high	[39,80]

**Table 4:** Unified semi-quantitative comparison of residual stress modeling approaches. (★★★★★ = excellent; ★★★★☆ = good; ★★★☆☆ = moderate; ★★☆☆☆ = limited; ★☆☆☆☆ = poor).

Criterion	Empirical	Analytical	FEM	Hybrid
Prediction accuracy	★★☆☆☆	★★★☆☆	★★★★★	★★★★☆
Computational efficiency	★★★★★	★★★★★	★☆☆☆☆	★★★★☆
Sensitivity robustness	★★☆☆☆	★★★☆☆	★★☆☆☆	★★★★☆
Scalability (multi-axis)	★★★☆☆	★★☆☆☆	★★★★★	★★★★☆
Physical interpretability	★☆☆☆☆	★★★★★	★★★★☆	★★★★☆
Industrial applicability	★★★★☆	★★★☆☆	★★☆☆☆	★★★★★

## Data Availability

No new data were created or analyzed in this study. Data sharing is not applicable to this article.
